# Concurrent Eosinophilic Esophagitis and Gastritis in an Adolescent: A Case Report and Literature Review

**DOI:** 10.7759/cureus.106219

**Published:** 2026-03-31

**Authors:** Bartosz Szarawarski, Anita Kuraś

**Affiliations:** 1 Pediatrics, Specialist Hospital No. 2, Bytom, POL

**Keywords:** chronic non-atrophic gastritis, eosinophilic esophagitis (eoe), eradication therapy, gastroduodenoscopy, helicobater pylori

## Abstract

Functional gastrointestinal disorders represent the most common cause of gastrointestinal complaints and do not require extensive diagnostic evaluation. However, the presence of so-called red flag symptoms, such as dysphagia or nocturnal abdominal pain, necessitates further investigation, including esophagogastroduodenoscopy (EGD). Eosinophilic esophagitis (EoE) is an increasingly recognized chronic, immune-mediated esophageal disorder. Its hallmark clinical manifestation is dysphagia, frequently presenting as food bolus impaction. In the pediatric population, symptoms may be nonspecific, and up to one-third of patients demonstrate no macroscopic abnormalities on endoscopic examination of the esophagus. Therefore, histopathological assessment of esophageal biopsy specimens remains the diagnostic gold standard (with an eosinophil count of ≥15 eosinophils per high-power field). First-line therapeutic options include proton pump inhibitors (PPIs), swallowed topical glucocorticosteroids, or dietary elimination therapy. These modalities demonstrate comparable efficacy, and treatment selection is typically based on shared decision-making between the clinician and the patient (and caregivers). Therapeutic response should be confirmed not only by clinical remission but also by normalization of both endoscopic and histological findings on follow-up evaluation. The disease is chronic, relapsing, and progressive if left untreated. Importantly, the presence of one gastrointestinal pathology does not preclude the coexistence of another. Chronic gastritis is a considerably more prevalent condition, and in children, it is often characterized by a paucity of symptoms despite its chronic course. Most cases are associated with infection by *Helicobacter pylori (H. pylori)*. Although *H. pylori* infection may remain asymptomatic, nausea and epigastric pain have been associated with its presence and may constitute early manifestations of chronic gastritis. In pediatric patients, detection of *H. pylori* infection in the absence of destructive gastric mucosal lesions does not necessitate eradication therapy. For more than two decades, accumulating evidence has suggested a potential inverse association between *H. pylori* infection and EoE; however, the underlying pathophysiological mechanisms of this relationship, if causal, remain incompletely elucidated. We present the case of a patient hospitalized in a pediatric department with a diagnosis of EoE and chronic gastritis. A unique feature of this clinical case is the coexistence of these two disease entities and the selection of an appropriate therapy that allows both conditions to be treated simultaneously.

## Introduction

Epigastric pain is one of the most common reasons for pediatric consultations in both outpatient and inpatient settings. In the adolescent population, abdominal pain, including pain localized to the epigastrium, occurs particularly frequently and may significantly affect psychosocial functioning, quality of life, and school attendance. The etiology of epigastric pain in adolescent girls is heterogeneous and includes both organic and functional causes. Functional disorders account for the vast majority of cases and, according to the Rome criteria, do not require extensive diagnostic evaluation [1]. However, a different approach is warranted in the presence of alarm symptoms from the so-called “red flag” list, including dysphagia [1]. In such cases, a comprehensive gastroenterological workup is indicated. For this reason, the described patient was admitted for inpatient evaluation. Although eosinophilic esophagitis (EoE) is not as common a condition as *Helicobacter pylori *(*H. pylori*) infection, gastritis, or gastroesophageal reflux disease (GERD), it has an established place in physicians’ diagnostic awareness. An association with allergic diseases was recognized relatively early [2]. Based on history and physical examination alone, it is not always possible to establish a diagnosis. It is noteworthy that even in the pediatric population, disorders of the same system may coexist-for example, GERD may cause epithelial dysfunction, thereby enabling antigenic stimulation, which constitutes one of the elements in the pathogenesis of EoE [2]. Gastroduodenoscopy with histopathological examination remains the fundamental and invaluable diagnostic tool in the presence of red-flag symptoms originating from the upper gastrointestinal tract [1,3]. 

## Case presentation

A 13-year-old girl was admitted to the Department of Pediatrics in Bytom because of epigastric pain, chest pain, and dysphagia. The swallowing problems had been present for several months. She described a feeling of pressure behind the sternum, episodes of food getting stuck in the esophagus, and choking when eating semisolid or solid foods. She also reported significant discomfort after meals, occasional nausea, and short episodes of pain between meals. Three months before admission, she had received pantoprazole for 14 days, prescribed by her primary care physician. The treatment led to only temporary improvement. The medical history did not indicate typical symptoms of GERD, such as heartburn, acid regurgitation, or a sensation of food content reflux after meals. Her medical history included recurrent headaches, chronic cough, and symptoms of chronic rhinitis; however, the patient had not been previously diagnosed with allergies. The family history was positive for atopic diseases. The girl’s father had bronchial asthma, and her brother had atopic dermatitis and an allergy to cow’s milk protein. On admission, her general condition was good. Physical examination showed reduced nasal patency with nasal discharge and tenderness in the precordial area on palpation. Laboratory tests revealed peripheral eosinophilia, markedly elevated total IgE level, and ketonuria. Laboratory findings are shown in Table 1. 

**Table 1:** Laboratory Findings on Admission WBC: white blood cells; RBC: red blood cells; MCV: mean corpuscular volume; MCH: mean corpuscular hemoglobin; MCHC: mean corpuscular hemoglobin concentration; ALT: alanine aminotransferase; AST: aspartate aminotransferase; CPK: creatine phosphokinase; CK-MB: creatine kinase myocardial band; CRP: C-reactive protein; TSH: thyroid-stimulating hormone

Parameter	Result	Reference range
WBC (×10^9/L)	7.5	4.0-10.0
Neutrophils (×10^9/L)	3.8	1.4-7.0
Neutrophils (%)	51.0	35.0-70.0
Lymphocytes (×10^9/L)	2.3	0.8-5.3
Lymphocytes (%)	31.2	20.0-53.0
Monocytes (×10^9/L)	0.6	0.0-1.2
Monocytes (%)	7.9	0.0-12.0
Eosinophils (×10^9/L)	0.7	0.0-0.7
Eosinophils (%)	8.7	0.0-7.0
Basophils (×10^9/L)	0.1	0.0-0.1
Basophils (%)	1.1	0.0-2.5
RBC (×10^12/L)	4.46	4.10-5.30
Hemoglobin (g/dL)	13.6	12.0-15.0
Hematocrit (%)	39.6	35.0-45.0
MCV (fL)	88.7	78.0-95.0
MCH (pg)	30.4	26.0-36.0
MCHC (g/dL)	34.3	32.0-36.0
Platelets (×10^9/L)	295	150-400
ALT (U/L)	11.1	0.0-33.0
AST (U/L)	22.1	0.0-38.0
CPK (U/L)	85.0	0.0-170.0
CK-MB (U/L)	18.5	0.0-25.0
CRP (mg/L)	0.99	0.0-5.0
Total IgE (IU/mL)	1522.0	0.0-200.0
Creatinine (mg/dL)	0.54	0.50-0.90
Magnesium (mmol/L)	0.82	0.65-1.07
Potassium (mmol/L)	4.19	3.50-5.10
Sodium (mmol/L)	136	135-145
Troponin T (pg/mL)	4.2	0.0-14.0
TSH (µIU/mL)	3.990	0.270-4.200

Specific IgE antibodies were positive to house dust mites, cat dander, and grass pollens. No food allergies were detected. The results of the allergy tests are presented in Table 2.

**Table 2:** Allergen-specific IgE results CCD: cross-reactive carbohydrate determinant IgE class interpretation: Class 0 (<0.35 kU/L), no detectable antibodies; Class 1 (0.35-<0.7), very low; Class 2 (0.7-<3.5), low; Class 3 (3.5-<17.5), moderate; Class 4 (17.5-<50), high; Class 5 (50-<100), very high; Class 6 (≥100), extremely high

Panel	Allergen	Class	IgE (kU/L)
Inhalant	Cypress pollen	1	0.44
Inhalant	Hazel pollen	0	0.18
Inhalant	Ash pollen	0	<0.15
Inhalant	Oak pollen	0	0.17
Inhalant	Olive pollen	0	<0.15
Inhalant	Birch pollen	0	<0.15
Inhalant	Rye pollen	0	0.16
Inhalant	Oat pollen	0	<0.15
Inhalant	Meadow grass pollen	0	0.29
Inhalant	Timothy grass pollen	2	2.9
Inhalant	Wheat pollen	0	<0.15
Inhalant	Orchard grass pollen	1	0.40
Inhalant	Bermuda grass pollen	0	<0.15
Inhalant	Plantain pollen	0	0.27
Inhalant	Goosefoot pollen	2	0.70
Inhalant	Pellitory pollen	0	0.24
Inhalant	Ragweed pollen	0	<0.15
Inhalant	Mugwort pollen	0	<0.15
Inhalant	Cat dander	4	18
Inhalant	Dog dander	0	0.20
Inhalant	Horse dander	0	0.18
Inhalant	Alternaria alternata	1	0.53
Inhalant	Aspergillus fumigatus	0	0.28
Inhalant	Candida albicans	1	0.42
Inhalant	Blomia tropicalis	2	0.79
Inhalant	Dermatophagoides pteronyssinus	6	>100
Inhalant	Dermatophagoides farinae	6	>100
Inhalant	Latex	0	<0.15
Inhalant	Cockroach	0	0.16
Inhalant	CCD/bromelain	0	<0.15
Food	Tomato	0	<0.15
Food	Avocado	0	<0.15
Food	Banana	0	0.21
Food	Citrus mix	0	0.17
Food	Kiwi	0	<0.15
Food	Peanut	0	<0.15
Food	Hazelnut	0	<0.15
Food	Pea	0	0.16
Food	Soy	0	<0.15
Food	Celery	0	<0.15
Food	Beef	0	0.26
Food	Chicken	0	<0.15
Food	Pork	0	0.16
Food	Mussel	0	0.22
Food	Shrimp	0	<0.15
Food	Crab	0	<0.15
Food	Tuna	0	<0.15
Food	Cod	0	<0.15
Food	Garlic	0	<0.15
Food	Onion	0	<0.15
Food	Yeast	0	<0.15
Food	Sesame	0	<0.15
Food	Rice	0	<0.15
Food	Corn	0	<0.15
Food	Wheat flour	0	<0.15
Food	Alpha-lactalbumin	0	<0.15
Food	Beta-lactoglobulin	0	0.16
Food	Casein	0	<0.15
Food	Egg white	0	<0.15
Food	CCD/Bromelain	0	<0.15

Parasitic infections were excluded through repeated stool examinations. Ultrasound of the neck and abdomen and chest X-ray were normal. In the initial diagnostic workup, pH monitoring for GERD was not performed, as the clinical history was not suggestive, and the test was considered potentially useful only in the absence of any specific abnormalities on endoscopic examination.

**Figure 1:**
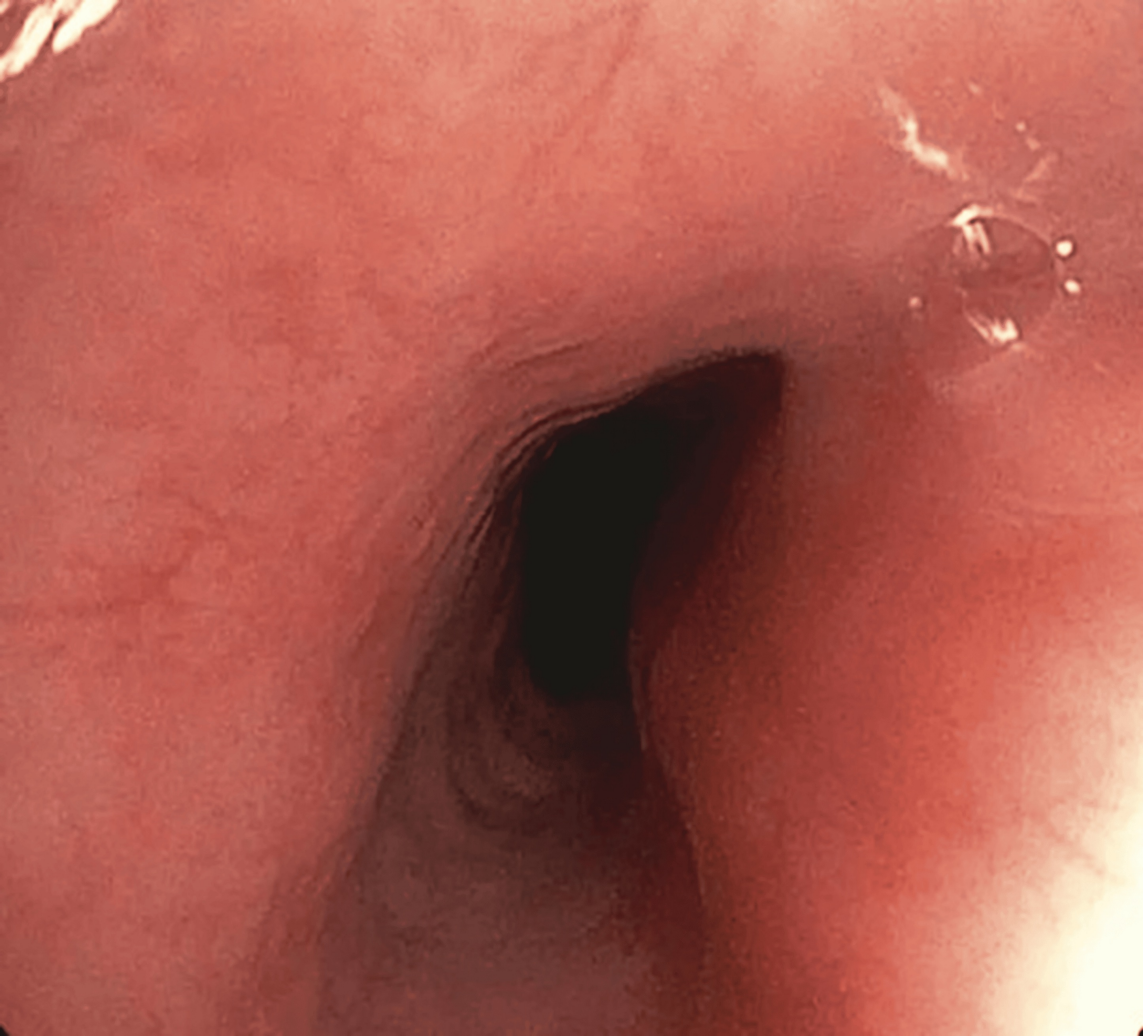
Endoscopic image of the proximal esophagus without macroscopically visible inflammatory changes

After the initial diagnostic workup, the most essential investigation for diagnostic completeness was performed, namely, upper gastrointestinal endoscopy with biopsy sampling. It showed superficial inflammation in the gastric body and antrum. The esophagus and duodenum appeared normal on macroscopic assessment. The esophageal image is presented in Figure 1.

Two biopsy specimens were obtained from the esophagus (proximal and distal parts), as well as three from the stomach, including one for a rapid urease test. No biopsies were taken from the duodenum. The rapid urease test was strongly positive, and *H. pylori *infection was confirmed on histopathology. The histopathological results were obtained almost two weeks after the procedure. Crucial for meeting the diagnostic criteria and determining the nature of the pathological process was the histopathological examination, which confirmed EoE and mild chronic active gastritis. The number of eosinophils in the gastric biopsy specimens did not indicate concomitant eosinophilic gastritis; however, the exact number was not specified in the report. The patient received a 14-day standard triple therapy for* H. pylori *eradication: omeprazole, amoxicillin, and metronidazole twice daily. After obtaining the complete set of investigations, proton pump inhibitor (PPI) therapy was continued for a total of eight weeks twice daily. Details of the medications, including formulations, dosages, and duration of therapy, are summarized in Table 3.

**Table 3:** Details of the applied treatment

Medication	Administration details
Omeprazole	40 mg twice daily orally for 14 days, followed by 20 mg twice daily for 6 weeks
Amoxicillin	1000 mg twice daily orally for 14 days
Metronidazole	500 mg twice daily orally for 14 days

When initiating PPI therapy, no dietary modifications were introduced. Only a healthy, balanced diet in accordance with general nutritional recommendations was advised. A follow-up endoscopy was planned after completion of treatment, but the patient did not return for hospitalization. During a telephone follow-up, her father reported a clear reduction in symptoms but admitted that the patient had not taken the medications regularly. He stated that she would remain under the care of an outpatient gastroenterology clinic.

## Discussion

EoE is a chronic immune- and antigen-mediated disorder characterized by symptoms of esophageal dysfunction resulting from a localized inflammatory infiltrate of the esophageal wall, predominantly composed of eosinophils [2-5]. Although EoE is not a common condition, it represents, alongside GERD, one of the most frequently diagnosed chronic esophageal disorders in both pediatric and adult populations [3,5]. Most reports in the literature demonstrate a male predominance, with a male-to-female ratio of up to 3:1. The disease also occurs more frequently in individuals with allergic conditions, with concomitant allergic rhinitis, asthma, atopic dermatitis, or urticaria reported in approximately 50-75% of cases [4,5]. In predisposed individuals, EoE is triggered by immune mechanisms primarily induced by food allergens (antigens). However, an association with inhalant allergens has also been demonstrated [2-5]. A proposed mechanism involves inhalation of allergens through the nasal and oral cavities, followed by their swallowing, or the generation of a regional immune response within the respiratory tract with subsequent migration of eosinophils to the esophagus [6]. In the immunopathogenesis of EoE, allergens induce a T helper 2 (Th2)-mediated immune response with the release of cytokines, including IL-4, IL-5, IL-13, and IL-33. This leads to activation of eosinophils, which produce eotaxins promoting and maintaining local esophageal inflammation [3,5]. The development of EoE is primarily driven by IgE-independent (delayed, cell-mediated) immune mechanisms, while IgE-dependent mechanisms appear to play a lesser role [3,6]. EoE is primarily driven by IgE-independent mechanisms with a lesser role for IgE-dependent pathways [3,6]. Identified risk factors for EoE include prematurity, cesarean section delivery, lack of or short duration of breastfeeding, antibiotic exposure in early childhood, and residence in sparsely populated areas [2,4,7]. 

The clinical presentation of EoE depends on the patient’s age, disease phenotype, and the ability of pediatric patients to describe their symptoms. Initially, the esophagus is affected predominantly by inflammation; however, with disease progression, EoE may evolve into a fibrostenotic phenotype characterized by esophageal remodeling and stricture formation [5,8]. In infants and young children, symptoms are often nonspecific and may include irritability, abdominal pain, feeding difficulties progressing to food refusal and growth impairment, as well as regurgitation and vomiting. In older children and adolescents, the predominant symptom is dysphagia, frequently accompanied by episodes of food impaction, heartburn, and chest pain [3,5,7,8]. There are no specific biomarkers that allow the diagnosis of EoE without endoscopic and histopathological evaluation. Mild peripheral eosinophilia is observed in approximately 5-50% of patients and is not pathognomonic, while elevated total IgE levels are reported in up to 70% of cases [3,6]. EoE may occur in both atopic and non-atopic individuals; therefore, diagnostic assessment based solely on specific IgE testing is not sufficiently reliable [2,3]. The diagnosis of EoE in children is based on endoscopic findings of the esophagus combined with histopathological examination of esophageal mucosal biopsies. Clinical symptoms do not correlate well with histologic disease activity [8]. Typical endoscopic features include esophageal rings (trachealization), linear furrows, white exudates or plaques, and esophageal strictures [3,6]. At least six mucosal biopsies should be obtained from a minimum of two different locations, usually the proximal and distal esophagus, due to the patchy distribution of lesions in EoE [2,5]. Biopsies should be taken regardless of the macroscopic appearance, as up to 30% of pediatric patients (and a smaller proportion of adults) may have a macroscopically normal esophagus [2,3]. The accepted diagnostic threshold is a peak eosinophil count of ≥15 eosinophils per high-power field (400× magnification), which provides a sensitivity of 100% and a specificity of 96% for the diagnosis of EoE [4,8]. Additional histologic features supporting the diagnosis include eosinophilic microabscesses, eosinophil degranulation, surface layering of eosinophils along the epithelium, dilated intercellular spaces, and basal zone hyperplasia and/or fibrosis [3]. EoE should be differentiated from other conditions associated with esophageal eosinophilia or overlapping clinical features, including GERD (typically ≤5 eosinophils per high-power field), gastrointestinal infections (fungal, parasitic, or viral), Crohn’s disease, connective tissue disorders, celiac disease, achalasia, drug-induced injury, hypereosinophilic syndrome, and eosinophilic inflammation involving other segments of the gastrointestinal tract (stomach or intestines) [3,6,8]. In a substantial proportion of patients, food bolus impaction is the predominant clinical manifestation. In such cases, contrast radiography of the upper gastrointestinal tract may be helpful, demonstrating contrast retention at the site of narrowing [3]. Unlike the focal distal esophageal strictures typically seen in GERD, strictures in EoE may be long-segment and difficult to detect endoscopically. Two studies demonstrated that 55% of pediatric patients with EoE had no visible stricture on endoscopy, while esophagography confirmed the presence of narrowing [4]. According to the most recent position statement of the European Society for Paediatric Gastroenterology, Hepatology and Nutrition (ESPGHAN), routine esophageal pH-impedance monitoring is not recommended in the diagnostic evaluation of EoE. However, this investigation may be useful in patients with concomitant GERD [2]. Patients often develop adaptive behavioral strategies to facilitate the swallowing of solid foods, such as avoiding certain food consistencies, cutting food into small pieces, adding fats or liquids, chewing excessively, and drinking large amounts of fluids with meals. Some children may also avoid eating in public settings. These compensatory behaviors may contribute to a delay in diagnosis [3-5].

There are three main goals in the treatment of esophagitis in children: histologic improvement with remission of inflammation to prevent esophageal remodeling and fibrosis, subjective relief of clinical symptoms, and restoration of normal growth and development, including appropriate anthropometric parameters [6]. Treatment efficacy should be monitored primarily by follow-up endoscopy with histologic assessment, as clinical improvement does not necessarily correlate with resolution of inflammation or prevention of fibrosis [3]. First-line therapy includes PPIs, topical corticosteroids, and elimination dietary therapy, which may also be used in combination. Each of these approaches may lead to remission. Therapeutic response should be evaluated endoscopically and histologically after at least 8-12 weeks of treatment. Multiple studies have demonstrated the effectiveness of PPI therapy in children. Clinical symptom resolution is achieved in more than 60% of patients, and histologic remission of esophageal eosinophilia in more than 50%. Due to regulatory approval, omeprazole is the most commonly used PPI in pediatric patients. The recommended dose is 1-2 mg/kg/day administered in two divided doses (maximum 40 mg twice daily). Twice-daily dosing has been shown to be more effective than once-daily administration [2,3,5,9]. Long-term management involves identifying the lowest effective maintenance dose to be used chronically. Discontinuation of PPI therapy is associated with recurrence of symptoms or esophageal eosinophilia within 3-6 months [8]. Topical corticosteroid therapy represents a second pharmacologic option and is effective in inducing both histologic and clinical remission in most patients. The two most commonly used agents are budesonide and fluticasone. In Poland, there are no formulations specifically registered for pediatric EoE treatment. Budesonide intended for nebulization may be swallowed during administration; however, greater efficacy is achieved when the medication is prepared as a viscous suspension to coat the entire length of the esophagus. This can be accomplished by mixing the drug with honey, agave syrup, applesauce, or sucralose. The recommended dose is 1-2 mg/day [2,3,5,6]. Fluticasone propionate may be administered from a metered-dose inhaler directly into the mouth (without a spacer) for swallowing while holding the breath. In children, induction doses range from 880 to 1760 µg/day, with maintenance therapy typically administered at approximately half of the induction dose [3]. The reported efficacy of topical corticosteroids in achieving clinical and histologic remission after 2-12 weeks of therapy ranges from 53% to 95% in various studies [4]. No significant difference in response between budesonide and fluticasone has been demonstrated in pediatric populations [2]. Medications should be administered at least 10 minutes after food or fluid intake, and patients should refrain from eating or drinking for 30-60 minutes after swallowing the medication [2,6]. An oral dispersible tablet formulation of budesonide is approved for adult patients with EoE. Topical corticosteroid therapy is generally safe and well-tolerated. The most common adverse effect is local candidiasis of the oral cavity and esophagus, occurring in 2-15% of patients, primarily during the induction phase and most often asymptomatic [2-4]. Other complications, such as adrenal suppression, bone demineralization, or growth impairment, are reported rarely [4]. Despite similar efficacy, systemic corticosteroids are not recommended because of a significantly higher risk of adverse effects [4]. Dietary therapy may also be used as a first-line treatment. Three main types of dietary elimination are applied: elemental diet, empirical elimination diet, and targeted elimination diet [6]. The elemental diet involves the complete elimination of all food allergens, with nutrition provided exclusively through amino acid-based formulas. Its efficacy reaches up to 90%. However, due to high financial costs and poor palatability, it is not widely used and is generally reserved for younger children and for older patients who fail to respond to an empirical elimination diet [2,3]. The most commonly used dietary intervention is the empirical elimination diet, which consists of excluding the most common food allergens. These include cow’s milk, eggs, fish and seafood, nuts, soy, and wheat. Elimination of all six allergens results in remission in approximately three-quarters of patients. Elimination of four allergens (cow’s milk, eggs, wheat, and soy) leads to remission in approximately 50-60% of patients. A two-food elimination diet (cow’s milk and wheat) may achieve adequate improvement in approximately 40% of cases [2,8]. The more restrictive the diet, the lower the patient adherence and the greater the potential negative impact on quality of life. Excessively restrictive elimination diets may adversely affect eating habits in children and may contribute to the development of avoidant/restrictive food intake disorder (ARFID) [2]. In a targeted elimination diet, foods are excluded based on positive skin prick tests or elevated specific IgE levels. However, the effectiveness of this approach is limited due to the insufficient diagnostic accuracy of allergy testing, particularly in non-atopic patients. In the pediatric population, the efficacy of targeted elimination diets does not exceed 50% [6]. Regardless of the type of dietary therapy used, patients should remain under the supervision of a dietitian during treatment [3]. If first-line therapy proves ineffective, combination treatment (dietary therapy plus pharmacologic therapy) may be considered. Other treatment options for EoE include endoscopic esophageal dilation, which is indicated in cases of significant luminal narrowing and severe dysphagia. However, dilation does not affect the underlying inflammatory process at the tissue level and does not prevent further disease progression [6]. Long-term untreated EoE may result in persistent symptoms related to structural and functional impairment of the esophagus. It is also associated with the risk of acute complications, including esophageal rupture during severe vomiting, aspiration pneumonia, and perforation related to endoscopic procedures [3].

A considerably more common condition with less specific clinical manifestations is gastritis. In its acute form, it is usually associated with an acute viral or bacterial infection and resolves within a few days. The most frequent cause of chronic gastritis in children is *H. pylori *infection, accounting for more than 90% of cases. Much less common causes include systemic stress, bile reflux, nonsteroidal anti-inflammatory drug use, Crohn’s disease, and eosinophilic inflammation [10]. Chronic gastritis is one of the most prevalent and insidious gastrointestinal disorders. It is estimated that up to half of the global population may be affected to some extent. The condition is typically a progressive, multistage process that, if untreated, may persist lifelong [11]. In* H. pylori *infection, the initial inflammatory changes are usually localized in the antral (pyloric) region of the stomach. The inflammatory infiltrate, composed predominantly of mononuclear cells, is initially superficial. However, the greater the cytotoxic potential of* H. pylori*, the more active the inflammation becomes, with the presence of neutrophilic infiltration. With prolonged disease duration, the process may assume a degenerative character [11]. Over time, inflammation often gradually extends from an antral-predominant form to involve the gastric body and fundus, resulting in pangastritis. Functional alterations of the stomach develop concurrently, primarily due to glandular atrophy of the mucosa and reduced gastric acid secretion. Loss of gastric glands in atrophic gastritis, which may develop in up to 50% of patients after many years, is followed by compensatory proliferation of immature intestinal-type glands. This process carries a risk of dysplasia and gastric carcinogenesis [11]. In the pediatric population, however, the most common endoscopic finding is nodularity of the gastric mucosa without features of atrophy or intestinal metaplasia. Histopathological examination typically confirms moderate chronic inflammation [12]. Clinical symptoms are often absent or mild and most commonly include nausea and epigastric pain [12]. More severe and highly symptomatic forms of chronic gastritis usually become clinically apparent in later decades of life [11].

*H. pylori* infection is considered one of the most common infections worldwide, with an estimated global prevalence of approximately 50% [12]. The prevalence is also high in the pediatric population, although, as in the general population, it varies geographically. Infection is most commonly acquired during childhood, and transmission occurs primarily via fecal-oral or oral-oral routes, with maternal-to-child transmission playing a significant role. The risk of infection increases in the presence of adverse socioeconomic factors, including household overcrowding, large family size, poor sanitary conditions, limited access to clean water, low educational level, and inappropriate dietary habits [12,13]. The infection is markedly more prevalent in developing countries. In high-income developed countries (including Poland), the prevalence has been steadily decreasing and is significantly lower than in low-income regions [12-14]. Up to 85% of* H. pylori *infections acquired in childhood are asymptomatic. When clinical manifestations occur, they are usually nonspecific, and some symptoms may be related to complications of infection, such as gastritis. A 2010 meta-analysis demonstrated a statistically significant positive association between *H. pylori *infection and nausea as well as epigastric pain. No significant association was found with other gastrointestinal symptoms, including vomiting, diarrhea, bloating, functional gastrointestinal disorders, halitosis, regurgitation, or constipation [15]. The ESPGHAN guidelines for the management of *H. pylori *infection in children and adolescents emphasize that the presence of the bacterium alone does not cause symptoms and do not recommend a “test-and-treat” strategy. The only exception is a positive family history of gastric cancer in a first-degree relative [14]. The primary indication for diagnostic evaluation for *H. pylori *infection is the presence of gastric and/or duodenal erosions or ulcers identified during endoscopy. Endoscopy itself is indicated in the presence of alarm symptoms suggesting an organic cause of significant upper gastrointestinal complaints, such as persistent vomiting, weight loss, or a positive family history of peptic ulcer disease [1,15]. Both invasive and noninvasive diagnostic methods for *H. pylori *infection are available, with varying sensitivity and specificity. Initial and definitive diagnosis should be based on invasive methods, whereas assessment of treatment response may be performed using noninvasive tests [13,14]. Current recommendations advise obtaining at least six gastric biopsies (three from the antrum and three from the body) for diagnostic purposes. Biopsy specimens should be evaluated using at least two methods: culture, molecular testing, or rapid urease test, together with histopathological examination. Infection is confirmed by a positive culture or molecular test, or by a positive rapid urease test in combination with positive histology. Whenever possible, antibiotic susceptibility testing should be performed, particularly for clarithromycin, due to the high resistance rates in children (even exceeding 50%). Routine testing for metronidazole susceptibility is not recommended because of limited reliability [14]. In pediatric patients, a 14-day eradication regimen is recommended, consisting of a PPI administered twice daily, high-dose amoxicillin, and a second antibiotic selected according to susceptibility results. If antibiotic susceptibility testing is unavailable, ESPGHAN recommends quadruple therapy including a PPI, amoxicillin, metronidazole, and bismuth. However, in Poland, bismuth-containing preparations are not approved for patients under 18 years of age. Therefore, many centers use triple therapy consisting of a PPI, amoxicillin, and metronidazole, with doses adjusted according to body weight. Sequential therapy is not recommended due to the increased risk of antibiotic resistance, microbiota disruption, and adverse effects. Treatment efficacy should be assessed approximately two months after completion of eradication therapy using noninvasive methods, primarily the monoclonal stool antigen test (SAT) [14]. Rapid immunochromatographic tests are not recommended because of their low sensitivity [14,16].

In the presented case, we decided to partially combine therapeutic strategies targeting both conditions. Considering the patient’s adolescent age and concerns regarding adherence to potential dietary recommendations, we initiated PPI therapy (omeprazole) as treatment for EoE for a minimum period of eight weeks, pending scheduled follow-up. This approach was also influenced by the concurrent decision to initiate eradication therapy for *H. pylori*. Although no erosions were observed during gastroscopy, the gastric mucosa demonstrated inflammatory changes, and the patient reported symptoms potentially attributable to* H. pylori *infection and gastritis, namely, nausea and epigastric pain. Despite the absence of antibiotic susceptibility testing, eradication therapy was based on a 14-day triple-drug regimen due to regulatory limitations regarding the use of bismuth salts in the pediatric population. A follow-up endoscopic examination was planned after two months of treatment; however, the patient did not attend. The legal guardian was informed by telephone about the necessity of continued gastroenterological follow-up due to the chronic nature of the disease. Over recent decades, *H. pylori* has attracted considerable scientific interest because of its pleiotropic clinical manifestations. Well-established etiological associations include chronic gastritis, peptic ulcer disease, gastric adenocarcinoma, and mucosa-associated lymphoid tissue (MALT) lymphoma. Beyond its well-documented pathogenic role in the stomach and duodenum, emerging data suggest a broader impact throughout the gastrointestinal tract, including possible associations with colorectal and esophageal malignancies. In addition,* H. pylori *infection has been linked to several extraintestinal manifestations. Confirmed associations include iron deficiency anemia, immune thrombocytopenia, and vitamin B12 deficiency, with less consistent evidence for dermatologic or metabolic disorders [17]. The literature predominantly suggests a potential “protective” effect of *H. pylori* infection against EoE, or at least an inverse association between the two conditions. The first study describing this inverse relationship was published in 2003 and involved an Australian pediatric population. Although the pathophysiology of EoE has not been fully elucidated, it is believed to involve multiple factors, primarily immunogenetic and environmental. *H. pylori* infection activates Th1 and Th17 immune responses while suppressing Th2-mediated pathways responsible for allergic reactions. In contrast, the pathogenesis of EoE involves a Th2-driven allergic inflammatory response, with regulatory T cells further modulating the Th1-Th2 balance. Similar to other allergic diseases, such as asthma, early *H. pylori* infection may promote a Th1-predominant immune profile and, to some extent, protect against Th2-mediated allergic responses and esophageal eosinophilia [17-19]. According to the hygiene hypothesis, reduced exposure to microorganisms in early childhood, resulting from improved sanitation, fewer infections, and urbanization, may lead to inadequate immune system conditioning and an increased risk of allergic diseases [20]. In a comprehensive review of the available literature, Doulberis et al. adopt a more critical perspective regarding the proposed protective role of *H. pylori* in EoE. They emphasize that many earlier studies had methodological limitations, including retrospective design, small sample sizes, lack of biopsy standardization, absence of PPI withdrawal prior to testing, and reliance on IgG serology for diagnosing *H. pylori* infection [17]. Moreover, the inverse relationship between the increasing incidence of EoE and the declining prevalence of *H. pylori *infection cannot be generalized globally, as these epidemiological trends are not uniform worldwide [17]. Overall, current immunological and epidemiological arguments appear simplified and incomplete. *H. pylori *infection should not be considered a potentially “beneficial” factor without robust evidence, particularly given its well-established role in inflammatory and neoplastic diseases [17,18].

## Conclusions

Despite the high prevalence of chronic gastritis, it usually does not produce severe or specific symptoms and is often diagnosed incidentally during endoscopic evaluation performed due to the presence of alarm symptoms. EoE should be primarily considered in patients presenting with dysphagia in its various clinical manifestations. Eosinophil quantification in biopsy specimens (> 15 eosinophils per high-power field as a diagnostic threshold) is essential and carries greater diagnostic significance than the macroscopic appearance of the esophagus. Given the availability of several first-line therapeutic options, treatment selection should be individualized and discussed with the patient in order to ensure optimal efficacy while minimizing the negative impact on quality of life. At the same time, careful analysis of the medical history and thorough clinical assessment are essential to avoid unnecessary interventions whenever possible. The greater the number of recommendations provided to the patient, the lower the likelihood of full adherence. It should also be emphasized that the presence of one disease entity does not exclude the coexistence of another, even in the context of reported inverse associations between certain conditions.
